# 

**Published:** 1858-11

**Authors:** 


*Editorial Courtesies.*—Since our advent into the editorial arena, we have been the recipient of many kind notices from the daily press. The daily papers of this city have kindly noticed each month the issue of our Journal, and many of the country papers have noticed us in a way which demands our warmest thanks. In spite of the hard times, our circulation is somewhat increased, and if subscribers would pay up, we should have no anxiety for the future. With the feeling that the Journal is not losing favor, and the kind commendations which we have occasionally received, the most tedious editorial duties become a pleasure. We hope never to deserve encouragement less, and trust that we may deserve it more than we do now.

---

*Back numbers of Journals.*—A correspondent is desirous of obtaining certain back numbers of several periodicals, as follows:

Am. Journal of Med. Sciences, old series, Nos. 1, 2, 5, 6, 35; new series, No. 9 of vol. X, and No. 13 of vol. VII.

New York Journal of Medicine, No. 1 of vol. I.

Transactions of American Medical Association, vol. IV.

Transactions of N. Y. State Med. Society for the years 1810, 1811, 1812, 1813, 1815, 1818, 1823.

Persons having any of these numbers, which they are willing to dispose of at a reasonable price, will confer a favor by addressing Dr. G. I. Fisher, Sing Sing, N. Y.

---

☞ Prof. Flint leaves to enter on the duties of his chair in the New Orleans School of Medicine, early in this month (November.) He expects to return to Buffalo in the latter part of February next. His correspondents, in the meantime, will please direct their letters to him at New Orleans.

